# Supplementary material for: Long-term monitoring projects of Brazilian marine and coastal ecosystems
Source: PeerJ. 2022 Nov 9;10:e14313. doi: 10.7717/peerj.14313 (PMC9653053; doi:10.7717/peerj.14313)
Supplement: Supplemental Information 1 — Variables monitored in each site from this study and other information. [file peerj-10-14313-s001.docx]

**Supplementary material**

**Diagnostics of long-term monitoring projects of marine and coastal environments in the Brazilian coast**

Cordeiro CAMM, Aued AW, Barros F, Bastos AC, Bender M, Mendes TC, Creed J, Cruz ICS, Dias MS, Fernandes LDA, Coutinho R, Gonçalves JEA, Floeter SR, Mello-Fonseca J, Freire AS, Gherardi DFM, Gomes LEO, Lacerda F, Martins RL, Longo GO, Mazzuco AC, Menezes R, Muelbert JH, Paranhos R, Quimbayo JP, Valentin JL, Ferreira CELF

**Table 1S**. Long-term ecological monitoring sites from PELD in Brazilian coastal environments with respective habitats monitored, frequency of monitoring and level of protection. MPA column indicates if the monitoring site is inside a Marine Protected Area, outside, or both.

| **PELD acronym** | **Project full name and website** | **Habitat monitored** | **MPA** | **Starting year as PELD project** |
| --- | --- | --- | --- | --- |
| Abrolhos | *Abrolhos*  *(http://abrolhos.org/a-rede/)* | Biogenic reef*, rhodolith bed, neritic zone, human socio-economic environment | Inside | 2006 |
| ELPA | *Estuário da Lagoa dos Patos e Costa Marinha Adjacente*  *(https://peld.furg.br/)* | Estuary, lagoon, neritic zone | Inside and outside | 1998 |
| CCAL | *Costa dos Corais Alagoas*  *(https://sites.google.com/view/peld-costa-dos-corais-al/)* | Biogenic reef, human socio-economic environment | Inside | 2016 |
| HCES | *Habitats Costeiros do Espírito Santo*  *(https://peldhces.wordpress.com)* | Estuary, biogenic reef, mangrove, rhodolith bed, rocky reef | Inside | 2016 |
| ILOC | *Monitoramento de Longa Duração das Comunidades Recifais das Ilhas Oceânicas Brasileiras*  *(https://peldiloc.sites.ufsc.br/)* | Biogenic reef*, rocky reef, neritic zone | Inside and outside | 2012 |
| PEBG | *Estrutura e Funções do ecossistema da Baía de Guanabara: uma Pesquisa Ecológica de Longa Duração*  *(http://www.peldguanabara.lncc.br)* | Estuary, mangrove, neritic zone, rocky shore, sandy beach, soft bottom | Inside and outside | 2008 |
| RECA | *Ressurgência de Cabo Frio*  *(https://www.marinha.mil.br/ieapm/peld)* | Rocky shore, rocky reef, neritic zone | Inside | 2016 |
| RLaC | *Restingas e Lagoas Costeiras do norte do Estado do Rio de Janeiro*  *(https://peld.macae.ufrj.br/)* | Lagoon, shore vegetation, sandy beach | Inside and outside | 2000 |
| TAMS | *Tamandaré Sustentável*  *(https://www.lecor.org/peld-tams)* | Biogenic reef*, estuary, human socio-economic environment, mangrove, neritic zone, seagrass, soft bottom | Inside | 2016 |

MPA – marine protected area; * – includes mesophotic reefs (> 40m deep).

**Table 2S**. Questions used in forms to evaluate attributes, characteristics and variables investigated by long-term monitoring of marine and coastal sites in Brazil.

| **Original version in Portuguese** | **English translation** |
| --- | --- |
| *Revisão sobre monitoramento temporal de ecossistemas marinhos do Brasil* | *Review of temporal monitoring of marine ecosystems in Brazil* |
| Abaixo seguem questões que necessitam de detalhamento para complementar a revisão proposta como o produto 1 do primeiro “Workshop de integração de séries temporais de ecossistemas marinhos do Brasil - Programa Ecológico de Longa Duração (PELD)” realizado em Arraial do Cabo em fevereiro de 2020, apoiado pela FAPERJ. | Below are questions that need further detail to complement the revision proposed as product 1 of the first "Workshop on the integration of time series of marine ecosystems in Brazil - Long Term Ecological Program (PELD)" held in Arraial do Cabo in February 2020, supported by FAPERJ. |
| **1) Detalhes do Projeto** | **1) Project Details** |
| Nome do pesquisador responsável pela série de dados do monitoramento: | Name of the researcher responsible for the monitoring data: |
| Instituição e laboratório/departamento do pesquisador responsável (indicar todas caso tenha trocado de instituição ao longo do período do monitoramento): | Institution and laboratory/department of the researcher responsible (indicate all if you have changed institutions during monitoring period): |
| Nome do programa de monitoramento: | Monitoring program name: |
| Ano de início do programa de monitoramento: | Monitoring program starting year: |
| O programa de monitoramento continua ativo?  sim ( ) não ( ) | Is the monitoring program still active?  Yes ( ) No ( ) |
| O monitoramento já sofreu interrupções ao longo de sua existência?  sim ( ) não ( ) | Has monitoring suffered any interruptions throughout its existence?  Yes ( ) No ( ) |
| Quais os motivos das interrupções?  Fim de financiamento ( )  Falta de recursos humanos ( )  Desastres naturais ( )  Outros ( ) | What were the reasons for the interruptions?  End of financing ( )  Lack of human resources ( )  Natural disasters ( )  Others ( ) |
| Se o monitoramento foi encerrado, qual o motivo?  Fim de financiamento ( )  Falta de recursos humanos ( )  Objetivos atingidos ( )  Outros ( ) | If monitoring was finished, for what reason?  End of financing ( )  Lack of human resources ( )  Objectives achieved ( )  Others ( ) |
| Caso continue ativo, existe previsão concreta (financiamento aprovado) para sua continuação?  ( ) sim ( ) não | If it remains active, is there a concrete forecast (approved financing) for its continuation?  ( ) Yes ( ) No |
| **2)** **Variáveis monitoradas e Unidades de conservação**  Para as questões com resposta discursiva, por favor, separar os itens usando " ; " (ponto-e-vírgula) | **2)** **Monitored Variables and Conservation Units**  For questions with a speech answer, please separate the items using " ; " (semicolon) |
| Quais são os habitats monitorados pelo programa (ex. recifes de corais, bancos de macroalgas ou de gramas marinhas, recifes profundos etc)? | What are the habitats monitored by the program (eg coral reefs, macroalgal or seagrass beds, deep reefs etc)? |
| Quais grupos biológicos são monitorados (ex. fitoplâncton, peixes, macrofauna, corais)? | Which biological groups are monitored (eg phytoplankton, fish, macrofauna, corals)? |
| Quais variáveis bióticas destes grupos são monitoradas (ex. abundância de peixes recifais, biomassa de fitoplâncton etc)? | What biotic variables from these groups are monitored (eg reef fish abundance, phytoplankton biomass etc)? |
| Qual a frequência de amostragem das variáveis (ex. diária, semanal, mensal, anual etc) bióticas? | What is the sampling frequency of biotic variables (eg, daily, weekly, monthly, annual etc)? |
| Quais variáveis abióticas são monitoradas, se alguma (ex. temperatura, salinidade, nitrato, granulometria etc)? | What abiotic variables are monitored, if any (eg temperature, salinity, nitrate, particle size, etc)? |
| O monitoramento é realizado dentro de Unidade de Conservação (UC)?  sim ( ) não ( ) Dentro e fora de UC ( ) | Is monitoring carried out within a Protected Area (PA)?  yes ( ) no ( ) Inside and outside of PA ( ) |
| Qual o nome da Unidade de Conservação e categoria? | What is the name of the Protected Area and its category? |
| Alguma dimensão ou variável social, econômica ou antrópica é monitorada? Qual(is)? | Is any social, economic or anthropic dimension or variable monitored? Which one(s)? |
| **3)** **Detalhamento dos financiamentos**  Nesta seção devem ser indicados o período e fontes de financiamento dos projetos de monitoramento | **3)** **Details of financing**  In this section should be indicate the period and sources of funding for monitoring projects |
| O programa é, ou já foi, associado ao programa Pesquisa Ecológica de Longa Duração (PELD) do CNPq?  sim ( ) não ( ) | Is the program, or has it ever been, associated with the CNPq's Long-Term Ecological Research Program (PELD)?  Yes ( ) No ( ) |
| O programa foi apoiado por mais de uma fonte financiadora ao longo do período de monitoramento? Detalhe abaixo. | Was the program supported by more than one funding source during the monitoring period? Detail below. |
| \|  \| CNPq \| FAPs \| Outras fontes \| Próprio pesquisador \| \| --- \| --- \| --- \| --- \| --- \| \| Único financiador \|  \|  \|  \|  \| \| < 10% do período \|  \|  \|  \|  \| \| 11% a 30% \|  \|  \|  \|  \| \| 31% a 50% \|  \|  \|  \|  \| \| > 51% \|  \|  \|  \|  \| | \|  \| CNPq \| FAPs \| Other sources \| Self funding \| \| --- \| --- \| --- \| --- \| --- \| \| Sole funder \|  \|  \|  \|  \| \| < 10% of the period \|  \|  \|  \|  \| \| 11% a 30% \|  \|  \|  \|  \| \| 31% a 50% \|  \|  \|  \|  \| \| > 51% \|  \|  \|  \|  \| |
| Se outras fontes de financiamento foram indicadas na pergunta anterior, por favor, detalhe abaixo indicando o nome e contribuição relativa de cada, caso seja mais de uma. | If other sources of funding were indicated in the previous question, please detail below indicating the name and relative contribution of each, if more than one. |
| Qual seria a somatória aproximada de todos os recursos já recebidos ou aprovados para o monitoramento ao longo de sua existência? Se possível, detalhe por financiador. | What would be the approximate sum of all resources already received or approved for monitoring throughout its existence? If possible, detail by funder. |
| **4)** **Interações, recursos humanos e produtos**  Nesta seção devem ser indicados detalhes dos níveis de interação intra e inter-institucionais dos programas de monitoramento, formação de recursos humanos, além de produtos gerados a partir do monitoramento. | **4)** **Interactions, human resources and products**  This section should indicate details of the levels of intra- and inter-institutional interaction of monitoring programs, training of human resources, in addition to the products generated from the monitoring. |
| Indique o número de pesquisadores associados ao programa de monitoramento (não incluir estudantes) ao longo de todo o período de existência. | Indicate the number of researchers associated with the monitoring program (do not include students) throughout its lifetime. |
| Indique, se houver, o nome de instituições (além da sede do programa) que são ou foram associadas ao programa de monitoramento ao longo de todo o período de existência. | Please indicate, if any, the name of institutions (other than the program headquarters) that are or have been associated with the monitoring program throughout its lifetime. |
| Indique o número de estudantes de pós-graduação associados ao programa de monitoramento ao longo de todo o período de existência, indicando número de mestrandos e doutorandos. | Indicate the number of graduate students associated with the monitoring program over the entire period of existence, indicating the number of master's and PhD students. |
| Indique o número de trabalhos de conclusão de curso, teses e dissertações (já desenvolvidas e em desenvolvimento) diretamente associadas ao programa de monitoramento. | Indicate the number of course completion papers, theses and dissertations (already developed and under development) directly associated with the monitoring program. |
| Indique o número de artigos científicos gerados a partir de dados do programa de monitoramento. | Indicate the number of scientific articles generated from the monitoring program data. |
| Existe algum produto gerado pelo grupo e associado ao monitoramento não citado aqui (e.g. livros, guias, documentários)? | Are there any products generated by the group and associated with monitoring not mentioned here (e.g., books, guides, documentaries)? |
| As atividades dos monitoramentos têm ou tiveram associação com atividade de ensino de graduação (e.g. aulas práticas)?  sim ( ) não ( ) | Do monitoring activities have, or did it have, association with undergraduate teaching activities (e.g., practical classes)?  Yes ( ) No ( ) |
| As atividades dos monitoramentos têm ou tiveram associação com atividades de ciência cidadã?  sim ( ) não ( ) | Do the monitoring activities have, or did it have, association with citizen science activities?  Yes ( ) No ( ) |
| O programa de monitoramento tem, ou teve, alguma estratégia de divulgação de suas atividades ou produtos? Detalhe abaixo. | Does the monitoring program have, or did it have, any dissemination strategy for its activities or products? Detail below. |
| \|  \| Sempre \| Esporadicamente \| Nunca \| \| --- \| --- \| --- \| --- \| \| Eventos e publicações científicas \|  \|  \|  \| \| Página de internet \|  \|  \|  \| \| Mídias sociais \|  \|  \|  \| \| Televisão \|  \|  \|  \| \| Rádio ou podcast \|  \|  \|  \| \| Jornal de grande circulação \|  \|  \|  \| \| Eventos à comunidade \|  \|  \|  \| \| Outros \|  \|  \|  \| | \|  \| Always \| Sometimes \| Never \| \| --- \| --- \| --- \| --- \| \| Scientific events and publications \|  \|  \|  \| \| Website \|  \|  \|  \| \| Social media \|  \|  \|  \| \| Television \|  \|  \|  \| \| Radio or podcast \|  \|  \|  \| \| Newspaper \|  \|  \|  \| \| Communitary Events \|  \|  \|  \| \| Others \|  \|  \|  \| |
| Como pode ser considerada a integração do programa de monitoramento com as políticas públicas locais? | How integrated is the monitoring program with local public policies? |
| Totalmente integrada ( )  Bem integrada ( )  Parcialmente integrada ( )  Não integrada ( ) | Fully integrated ( )  Well integrated ( )  Partially integrated ( )  Not integrated ( ) |
| **5)** **Observações e complementos**  Agradecemos pelo tempo empenhado em responder este questionário. O produto gerado a partir dele será de grande importância para o fortalecimento e aperfeiçoamento dos programas de monitoramento em ambientes marinhos brasileiros. | **5)** **Observations and additional information**  Thank you for taking the time to answer this questionnaire. The product generated from it will be of great importance for the strengthening and improvement of monitoring programs in Brazilian marine environments. |
| Caso tenha alguma contribuição que queira adicionar ou questão não abordada anteriormente, por favor, indique abaixo. | If you have any contribution you would like to add or a question that has not been addressed previously, please indicate below. |

**Table 3S.** Partner institutions of long-term monitoring projects (PELD) in the Brazilian coast until January 2020.

| **Code** | **Institution** | **Type** | **PELD partner** |
| --- | --- | --- | --- |
| 1 | Centro Nacional de Conservação da Biodiversidade Marinha do Nordeste (CEPENE)/Brazil | governmental institution | TAMS |
| 2 | Costa Semi-Árida do Brasil PELD/Brazil | PELD project | ELPA |
| 3 | Empresa Brasileira de Pesquisa Agropecuária (EMBRAPA)/Brazil | governmental institution | RLaC |
| 4 | Environment Agency/Austria | foreign institution | RLaC |
| 5 | Universidade Federal do Rio Grande (FURG)/Brazil | Brazilian university | ELPA, RLaC |
| 6 | Goethe Universität Frankfurt/Germany | foreign institution | RLaC |
| 7 | Instituto Brasileiro do Meio Ambiente e Recursos Naturais Renováveis (IBAMA)/Brazil | governmental institution | TAMS |
| 8 | Instituto Chico Mendes de Conservação da Biodiversidade (ICMBio)/Brazil | governmental institution | Abrolhos, CCAL, ELPA, RECA, TAMS |
| 9 | Instituto de Estudos do Mar Almirante Paulo Moreira (IEAPM)/Brazil | governmental institution | RECA |
| 10 | Instituto Nacional de Pesquisas Espaciais (INPE)/Brazil | governmental institution | Abrolhos, ILOC, PEBG |
| 11 | Jardim Botânico do Rio de Janeiro/Brazil | governmental institution | Abrolhos |
| 12 | KAOSA ONG/Brazil | NGO | ELPA |
| 13 | Laboratório Nacional de Computação Científica/Brazil | Brazilian university | PEBG |
| 14 | Ministério da Agricultura, Pecuária e Abastecimento/Brazil | governmental institution | ELPA |
| 15 | Ministério de Ciências, Tecnologia e Inovação/Brazil | governmental institution | ELPA |
| 16 | Núcleo de Estudos e Monitoramento Ambiental (NEMA – Rio Grande)/Brazil | governmental institution | ELPA |
| 17 | National Oceanic and Atmospheric Administration (NOAA)/USA | foreign institution | ELPA |
| 18 | ONG Biota/Brazil | NGO | CCAL |
| 19 | Oregon State University/USA | foreign institution | HCES |
| 20 | Portos RS/Brazil | governmental institution | ELPA |
| 21 | Pontifícia Universitária Católica - Rio de Janeiro (PUC-Rio)/Brazil | Brazilian university | PEBG |
| 22 | Texas A&M University/USA | foreign institution | ELPA |
| 23 | The University of Adelaide/Australia | foreign institution | ELPA |
| 24 | Universidade Estadual de Maringá (UEM)/Brazil | Brazilian university | RLaC |
| 25 | Universidade Estadual do Rio de Janeiro (UERJ)/Brazil | Brazilian university | ELPA, PEBG |
| 26 | Universidade Estadual de Santa Cruz (UESC)/Brazil | Brazilian university | TAMS |
| 28 | Universidade Federal do Ceará (UFCE)/Brazil | Brazilian university | ELPA |
| 29 | Universidade Federal Fluminense (UFF)/Brazil | Brazilian university | Abrolhos, HCES, ILOC, PEBG, RECA |
| 30 | Universidade Federal de Pernambuco (UFPE)/Brazil | Brazilian university | Abrolhos, CCAL, ELPA |
| 31 | Universidade Federal do Paraná (UFPR)/Brazil | Brazilian university | ELPA, HCES, ILOC |
| 32 | Universidade Federal do Rio Grande do Sul (UFRGS)/Brazil | Brazilian university | Abrolhos, ELPA, RLaC |
| 33 | Universidade Federal do Rio de Janeiro (UFRJ)/Brazil | Brazilian university | ILOC, PEBG, RLaC |
| 34 | Universidade Federal do Rio Grande do Norte (UFRN)/Brazil | Brazilian university | ILOC, PEBG |
| 35 | Universidade Federal Rural de Pernambuco (UFRPE)/Brazil | Brazilian university | TAMS |
| 36 | Universidade Federal Rural do Rio de Janeiro (UFRRJ)/Brazil | Brazilian university | PEBG |
| 37 | Universidade Federal do Sul da Bahia (UFSB)/Brazil | Brazilian university | Abrolhos |
| 38 | Universidade Federal de Santa Catarina (UFSC)/Brazil | Brazilian university | HCES, ILOC |
| 39 | Universidade Federal de Santa Maria (UFSM)/Brazil | Brazilian university | ILOC |
| 40 | Universidade Estadual Paulista (UNESP)/Brazil | Brazilian university | RLaC |
| 41 | Universidade Federal do Estado do Rio de Janeiro (UNIRIO)/Brazil | Brazilian university | PEBG, RECA |
| 42 | Universidade do Sul de Santa Catarina (UNISUL)/Brazil | Brazilian university | HCES |
| 43 | Universidad de la República/Uruguay | foreign institution | RLaC |
| 44 | Universidad Nacional de Mar del Plata/Argentina | foreign institution | RLaC |
| 45 | Universidade Luterana do Brasil/RS/Brazil | Brazilian university | RLaC |
| 46 | University of Kiel/Germany | foreign institution | RLaC |
| 47 | University of North Texas/USA | foreign institution | ELPA |
| 48 | University of South Carolina/USA | foreign institution | ELPA |
| 49 | University of Southern California/USA | foreign institution | PEBG |
| 50 | Universidade de São Paulo (USP)/Brazil | Brazilian university | HCES, ILOC, PEBG, RLaC, TAMS |
| 51 | Yaqu Pacha/Germany | NGO | ELPA |

ILOC – *Monitoramento de Longa Duração das Comunidades Recifais das Ilhas Oceânicas Brasileiras*, TAMS – *Tamandaré Sustentável*, CCAL - *Costa dos Corais Alagoas*, HCES - *Habitats Costeiros do Espírito Santo*, RLaC - *Restingas e Lagoas Costeiras do norte do Estado do Rio de Janeiro*, RECA – *Ressurgência de Cabo Frio*, PEBG - *Estrutura e Funções do ecossistema da Baia de Guanabara*, ELPA – *Estuário da Lagoa do Patos e Áreas Adjacentes*.

**Table 4S.** Human resources formed and scientific and social production of PELD sites research groups until January 2020.

| **PELD site** | **Number of students** | **Type of publication** | | | | | | | |
| --- | --- | --- | --- | --- | --- | --- | --- | --- | --- |
|  |  | **Thesis** | **Articles** | **Book** | **Book chapter** | **Protocol** | **Video** | **Game** | **Cartoon** |
| PELD-Abrolhos | 35 | 35 | 60 | 0 | 0 | 0 | 1 | 0 | 0 |
| PELD-CCAL | 19 | 19 | 6 | 0 | 0 | 0 | 0 | 0 | 1 |
| PELD-ELPA | 106 | 171 | 256 | 3 | 63 | 0 | 0 | 0 | 0 |
| PELD-HCES | 4 | 9 | 8 | 0 | 0 | 1 | 0 | 0 | 0 |
| PELD-ILOC | 31 | 10 | 15 | 0 | 1 | 0 | 0 | 0 | 0 |
| PELD-PEBG | NI | 32 | 93 | 2 | 3 | 0 | 0 | 0 | 0 |
| PELD-RECA | 15 | 15 | 20 | 0 | 0 | 0 | 0 | 1 | 0 |

**Table 5S.** List of monitored habitats, indicators, variables or taxonomic groups and frequency of monitoring of long-term monitoring projects (PELD) in the Brazilian coast until January 2020.

| **PELD site** | **Habitat type** | **Monitored indicators, variables or taxonomic groups** | **Frequency** |
| --- | --- | --- | --- |
| PELD-Abrolhos | biogenic reef | Resources and socio-economic | annual |
| PELD-Abrolhos | biogenic reef | Ichthyofauna | annual |
| PELD-Abrolhos | biogenic reef | Benthic organisms | annual |
| PELD-Abrolhos | biogenic reef | Macrofauna (> 0.5 cm) | annual |
| PELD-Abrolhos | biogenic reef | Scleractinia | annual |
| PELD-Abrolhos | water column | Ichthyofauna | annual |
| PELD-Abrolhos | biogenic reef | Reef accretion | annual |
| PELD-CCAL | biogenic reef | Resources and socio-economic | annual |
| PELD-ELPA* | coastal lagoon | Phytoplankton | monthly |
| PELD-ELPA* | water column | Zooplankton | monthly |
| PELD-ELPA* | estuary | Primary producers and consumers | seasonal |
| PELD-ELPA* | unconsolidated bottom | Macrofauna (> 0.5 cm) | seasonal |
| PELD-ELPA* | estuary | Submersed vegetation | monthly |
| PELD-ELPA* | estuary | Submersed vegetation | quarterly |
| PELD-ELPA* | water column | Phytoplankton | monthly |
| PELD-ELPA* | water column | Ichthyofauna | monthly |
| PELD-ELPA* | water column | Ichthyoplankton | monthly |
| PELD-ELPA* | water column | *Tursiops truncatus* | fortnightly |
| PELD-HCES | biogenic reef | Benthic cover | monthly |
| PELD-HCES | biogenic reef | eDNA | monthly |
| PELD-HCES | rhodolith beds | Ichthyofauna | annual |
| PELD-HCES | rhodolith beds | Benthic cover | annual |
| PELD-HCES | rhodolith beds | eDNA | annual |
| PELD-HCES | estuary | Macrofauna (> 0.5 cm) | quarterly |
| PELD-HCES | mangrove | Carbon stock | annual |
| PELD-HCES | mangrove | Leaf litter | monthly |
| PELD-ILOC | rocky reef | Ichthyofauna | annual |
| PELD-ILOC | rocky reef | Scleractinia and symbionts | annual |
| PELD-ILOC | rocky reef | Benthic cover | annual |
| PELD-ILOC | rocky reef | *Grapsus grapsus* | biannual |
| PELD-ILOC | rocky reef | *Grapsus grapsus* | annual |
| PELD-ILOC | rocky reef | *Montastrea cavernosa* | annual |
| PELD-ILOC | rocky reef | *Siderastrea* spp. | quarterly |
| PELD-ILOC | rocky reef | *Siderastrea* spp. | annual |
| PELD-ILOC | rocky reef | *Siderastrea* spp. | biannual |
| PELD-ILOC | rocky reef | *Montastrea cavernosa* | biannual |
| PELD-ILOC | rocky reef | Benthic cover | monthly |
| PELD-ILOC | rocky reef | Echinoidea abundance | annual |
| PELD-PEBG | water column | Bacterioplankton | annual |
| PELD-PEBG | hydrology | Water parameters | annual |
| PELD-PEBG | rocky reef | Benthic cover | annual |
| PELD-PEBG | sandy beach | Macrofauna (> 0.5 cm) | annual |
| PELD-PEBG | water column | Ichthyofauna | annual |
| PELD-PEBG | unconsolidated | Macrofauna (> 0.5 cm) | annual |
| PELD-PEBG | mangrove | Vegetation | annual |
| PELD-RECA | water column | Meroplankton | weekly |
| PELD-RECA | water column | Holoplankton | weekly |
| PELD-RECA | water column | Phytoplankton | weekly |
| PELD-RECA | water column | Bacterioplankton | weekly |
| PELD-RECA | water column | Phytoplankton | annual |
| PELD-RECA | water column | Zooplankton | annual |
| PELD-RECA | rocky reef | benthic cover | quarterly |
| PELD-RECA | rocky reef | Ichthyofauna | quarterly |
| PELD-RECA | intertidal | Benthic cover | quarterly |
| PELD-RLaC | coastal lagoon | *Didelphis aurita* | quarterly |
| PELD-RLaC | coastal lagoon | *Philander quica* | quarterly |
| PELD-RLaC | coastal lagoon | *Cerradomys goytaca* | quarterly |
| PELD-RLaC | coastal lagoon | *Atlantorchestoidea brasiliensis* | quarterly |
| PELD-RLaC | coastal lagoon | *Atlantorchestoidea brasiliensis* | annual |
| PELD-RLaC | coastal lagoon | *Atlantorchestoidea brasiliensis* | bimonthly |
| PELD-RLaC | coastal lagoon | *Quadrivisio lutzi* | annual |
| PELD-RLaC | coastal lagoon | *Quadrivisio lutzi* | quarterly |
| PELD-RLaC | coastal lagoon | Ichthyofauna | quarterly |
| PELD-RLaC | coastal lagoon | Ichthyofauna | biannual |
| PELD-RLaC | coastal lagoon | Ichthyofauna | annual |
| PELD-RLaC | coastal lagoon | Vegetation (*Clusia* sp.) | monthly |
| PELD-RLaC | coastal lagoon | Vegetation | fortnightly |
| PELD-TAMS | biogenic reef | Macroinvertebrates | biannual |
| PELD-TAMS | biogenic reef | Ichthyofauna | biannual |
| PELD-TAMS | biogenic reef | Macrofauna (> 0.5 cm) | biannual |
| PELD-TAMS | biogenic reef | Scleractinia | biannual |
| PELD-TAMS | estuary | Ichthyofauna | biannual |
| PELD-TAMS | estuary | Macrofauna (> 0.5 cm) | biannual |
| PELD-TAMS | mesophotic reef | Ichthyofauna | biannual |
| PELD-TAMS | mesophotic reef | Macrofauna (> 0.5 cm) | biannual |
| PELD-TAMS | mesophotic reef | Scleractinia | biannual |
| PELD-TAMS | seagrass bed | Seagrass | annual |
| PELD-TAMS | biogenic reef | Macroalgae | seasonal |
| PELD-TAMS | unconsolidated substrate | Macroalgae | annual |
| PELD-TAMS | water column | Phytoplankton | annual |
| PELD-TAMS | water collumn | Zooplankton | annual |
| PELD-TAMS | water column | Ichthyoplankton | annual |
| PELD-TAMS | estuary | Ichthyofauna | quarterly |
| PELD-TAMS | estuary | Macroinvertebrates | biannual |
| PELD-TAMS | estuary | Primary producers | biannual |
| PELD-TAMS | estuary | Organic matter | biannual |
| PELD-TAMS | biogenic reef | Resources and socio-economic | annual |

* More details about PELD ELPA monitoring can be found in Lemos *et al*. (2022) Patos Lagoon estuary and adjacent marine coastal biodiversity long-term data. Earth Syst. Sci. Data, 14, 1015–1041. DOI: 10.5194/essd-14-1015-2022
